# Ketogenic diet may be a new approach to treatment stress urinary incontinence in obese elderly women: report of five cases

**DOI:** 10.1186/s12905-022-01987-5

**Published:** 2022-10-04

**Authors:** Yu Sun, Haixia Chen, Yueran Bai, Tingyue Zhang, Wenpei Bai, Bo Jiang

**Affiliations:** 1 Department of Obstetrics and Gynecology, Beijing Shijitan Hospital, Capital Medical University, Beijing, 100038 China; 2 Department of Obstetrics and Gynecology, Beijing Huimin Hospital, Beijing, China; 3 Department of Nutrition, Aviation General Hospital, Beijing, China; 4 Xiangya Medical College, Central South University, Changsha, China; 5 Department of Neurosurgery, Beijing Tiantan Hospital, Capital Medical University, Beijing, China

**Keywords:** Stress urinary incontinence, Ketogenic diet, Urine leakage, Obese

## Abstract

**Background:**

Stress urinary incontinence (SUI) as a serious social problem restricted women's daily life and affect their quality of life, especially for obese women. The mechanism of stress urinary incontinence is unclear. Weight loss is the first line of treatment for stress incontinence in obese women. Ketogenic diet is a special diet with high fat, low carbohydrate and moderate protein, which can reduce body mass faster than the traditional diet. There exist no reports on the therapeutic effect of ketogenic diet on SUI in obese women.

**Case presentation:**

Five postmenopausal obese women are diagnosed as mild to moderate stress urinary incontinence, which affected their quality of life for medical treatment. After 4 weeks ketogenic diet, we found that ketogenic diet can significantly improve urine leakage, reduce body weight, decrease visceral fat area, reduce body fat percentage, and reduce BMI.

**Conclusion:**

Reports in this case reveal that ketogenic diet may become one of the effective methods for the treatment of stress urinary incontinence in obese women in the future, providing a minimally invasive, highly profitable and highly compliant treatment for stress urinary incontinence in obese women.

## Background

Obesity is a chronic disease with high prevalence that is difficult to manage [1]. As we know, obesity causes functional disabilities, reduced quality of life and reduced life expectancy, and is known to contribute to increases in chronic diseases, including cerebrovascular and cardiovascular diseases, diabetes, sleep apnea and pelvic floor dysfunctions [2, 3]. Stress urinary incontinence (SUI) as a serious social problem restricted women’s daily life and affected their quality of life. Epidemiological data show that the overall incidence of SUI was 18.9% in women, with the age of onset increasing with age, and the highest incidence of SUI was 28.2% in the 50–59 age group [4]. Risk factors for SUI include age, genetic factors, decreased estrogen, constipation, chronic cough, obesity, etc. [5, 6]. The mechanism of stress urinary incontinence has been reported. Related hypotheses include: pressure conduction theory, high urethral motility theory, internal urethral sphincter defect theory and hammock hypothesis. However, none of them is widely accepted. More and more scholars believe that the occurrence of SUI is the result of interaction of multiple factors. Treatments for stress incontinence in women is varied, including conservative treatment and surgical treatment, but the survey shows that most women prefer the conservative treatment. Weight loss is the first line of treatment for stress incontinence in obese women [7, 8]. Ketogenic diet is a special diet with high fat, low carbohydrate and moderate protein, which can reduce body mass faster than the traditional diet. These cases are the first attempt to treat SUI in obese women with ketogenic diet, and the effect was encouraging. It provide a new direction and idea for the treatment of stress urinary incontinence in obese patients and look forward to become a new method for the treatment of stress urinary incontinence in obese women.

## Cases presentation

### Selection criteria of subjects and intervention

The participants inclusion criteria were as follows: (1) 40–60 years old; (2)BMI ≥ 28 kg/m^2^; (3) Sneeze, cough, laugh or exercise, involuntary urine leaks from the urethral orifice; (4) One hour urine pad test ≥ 2 g; (5) Subjects voluntarily participated in this study and sign the informed consent form. Exclusion criteria: (1) Urodynamic examination excludes urgent urinary incontinence and mixed urinary incontinence; (2) History of urinary incontinence or pelvic floor surgery; History of total hysterectomy or subtotal hysterectomy; (3) History of pelvic radiotherapy; (4) Pelvic organ prolapse; (5) Urinary infection; (6) One hour urine pad test ≥ 10 g; (7) Other methods are being used to treat stress urinary incontinence; (8) Complicated with abnormal functions of important organs such as heart, liver, kidney and hematopoietic system.

The intervention was as shown in the previous study [9], and the details were as follows:The energy of ketogenic diet is about 5–10% of carbohydrate (≤ 50 g/day), 18–27% from protein and 70–75% from fat. The daily calories is about 1300–1500 kcal/day, which were calculated by a qualifified dietitian based on the basic metabolic rate of the women’s body composition analysis. Daily calorie demand is assigned to the menu of three meals, provided to participants through mobile phones and updated every 3 days. During the 4-week intervention, all participants were contacted by mobile phone to ensure compliance and to address any issues. Compliance with the dietary regimen was monitored by taking daily measurements of urinary ketones. One hour pad test is an objective index to evaluate stress urinary incontinence, which has been widely used in previous studies [10, 11]. After 4-week KD treatment, 1-h urine pad test was used to evaluate urine leakage.

### Case 1

**Fig. 1:**
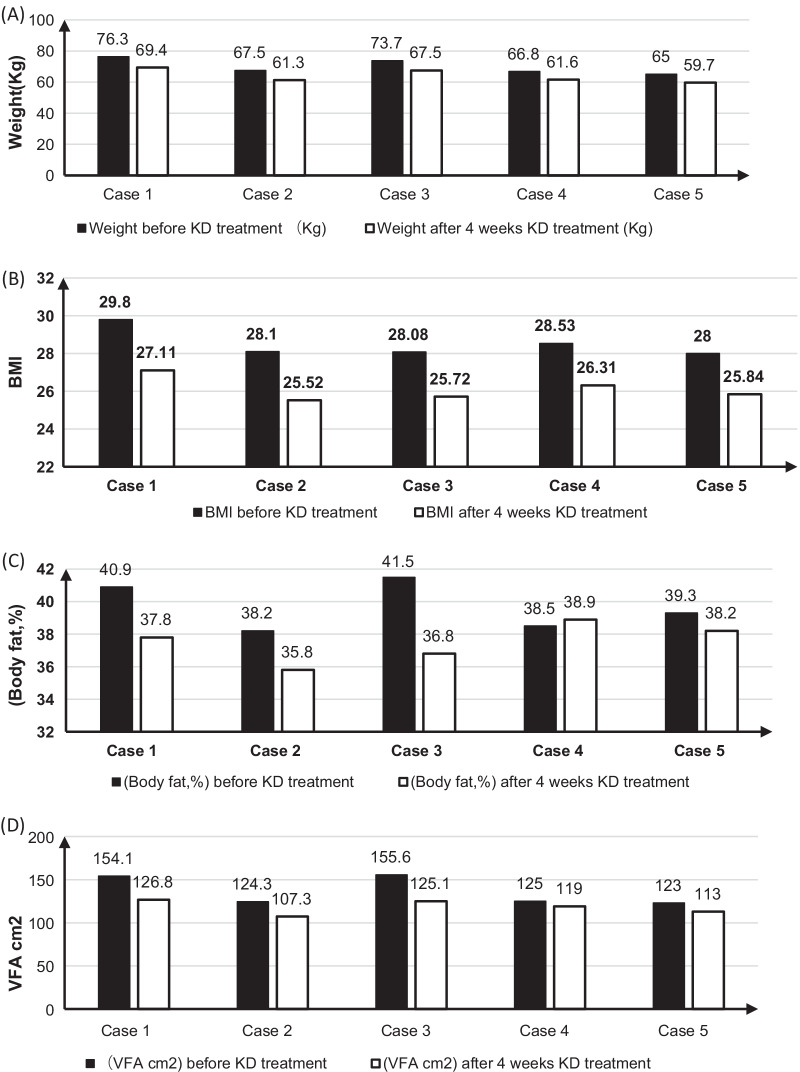
Analysis of human body composition before and after treatment. **A** Comparison of body weight before and after ketogenic diet treatment. **B** Comparison of BMI before and after ketogenic diet treatment. **C** Comparison of body fat percentage before and after ketogenic diet treatment. **D** Comparison of visceral fat area before and after ketogenic diet treatment

**Fig. 2:**
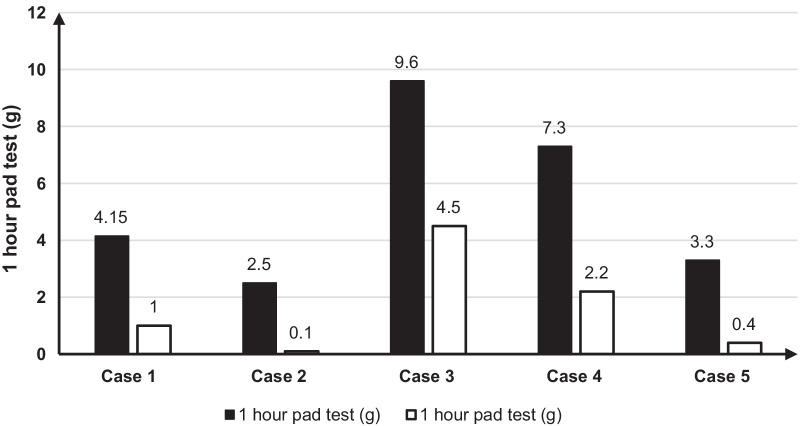
One hour pad test before and after treatment

A 52‐year‐old woman presented to our hospital with a 23-year history of urine leakage, which gradually worsened over a year. Twenty-three years ago, after vaginal delivery, she developed the symptoms of urine leakage after severe cough, sneezing, without the symptoms of frequent, urgent and painful urination. Nearly 1 year, leakage of urine after slight cough and sneeze, which affect her normal life. She came to the hospital for further treatment. Physical examination: height: 160 cm, weight: 76.3 kg. Weight has increased significantly in recent 10 years. BMI: 29.8. An hour pad test: 4.15 g. On vaginal examination with no anterior or posterior vaginal wall or uterine prolapse. And Kupperman score was 37 points. After 4 weeks of ketogenic diet treatment, the main results are shown in the Figs. 1 and 2 and Table 1 below. The weight loss is 6.9 kg, the percentage of body fat decrease by 3.1%, and proportion of body skeletal muscle increase 1.62%. BMI decrease 2.69 and visceral fat area reduce 27.3 cm^2^. Self-conscious leakage of urine was improved compared to before treatment. After 1-h pad test: 1 g. Menopausal symptoms were obviously improved, and kupperman score decrease from 37 to 15 points, especially insomnia and urinary tract infection obviously improved.

**Table 1:** Laboratory examinations before and after treatment

	Before the KD treatment (case 1)	4 weeks after KD treatment (case 1)	Before the KD treatment (case 2)	4 weeks after KD treatment(case 2)	Before the KD treatment (case 3)	4 weeks after KD treatment(case 3)	Before the KD treatment (case 4)	4 weeks after KD treatment(case 4)	Before the KD treatment (case 5)	4 weeks after KD treatment(case 5)
White blood cells(10^9/L)	11.97	5.46	6.42	5.37	5.97	4.81	5.8	4.8	5.9	5.3
Hemoglobin(g/L)	142	139	153	131	126	138	130	148	134	142
Platelet(10^9/L)	231	219	330	227	198	220	209	164	231	148
Urine leukocyte(HF)	45.42	182.46	4.91	0	0	0	0	0	0	0
Urine red blood cells(HF)	0	16.67	0	77.97	29.83	0	0	0	0	0
Urine ketone	–	2 +	–	3 +	–	2 +	–	3 +	–	3 +
Urine protein	–	–	–	–	–	–	–	–	–	–
Ery	–	–	–	-	3 +	3 +	–	–	–	–
AST (U/L)	51	34	30	34	14	16	20.32	13	19.7	14.9
ALT (U/L)	86	24	34	34	11	16	15.22	18.79	19.5	18.05
Serum glucose (mmol/L)	5.34	5.25	5.91	5.01	4.93	5.33	5.37	4.6	5.1	4.11
Cholesterol (mmol/L)	5.38	5.56	5.22	5.28	5.59	5.45	5.46	5.9	5.23	4.57
Triglycerides (mmol/L)	1.78	0.94	1.04	0.81	1.23	0.95	1.07	0.73	1.42	0.84
HDLC (mmol/L)	1.05	1.03	1.2	1.09	1.41	1.28	1.45	1.28	1.39	1.28
LDLC (mmol/L)	3.94	4.01	3.16	3.39	3.27	3.29	3.24	3.8	3.23	2.79
Albumin (g/L)	49.4	40.7	45.8	38	45.1	46.7	42.45	44.42	42.5	43.95
Uric acid (umol/L)	456	562	321	417	290	336	287.6	350.4	319.91	464.1
Creatinine (umol/L)	59	73	50	47	53	54	49.16	48.41	53.1	45.9

### Case 2

A 51-year-old postmenopausal woman was diagnosed with stress urinary incontinence for 20 years. In 1999, she delivered a baby, weighing 3,500 g. She had a smooth vaginal delivery without forceps. Leaky urine is accompanied by coughing, sneezing and walking quickly after delivery. No symptoms such as frequent urination, urgent urination and painful urination were found, and no treatment was given. Two years ago, she realized that the symptoms of urine leakage became worse after her rapid weight gain. An hour pad test: 2.5 g. Height: 155 cm, Weight: 67.5 kg, BMI: 28.1.

Pelvic examination: no obvious prolapses were observed in uterine and vaginal walls. Kupperman score: 26 points. Treatment: the ketogenic diet was described as before. After 4 weeks of ketogenic diet treatment, the main results are shown in the Figs. 1 and 2 and Table 1 below. The weight loss is 6.2 kg, the percentage of body fat decrease by 2.7%, and proportion of body skeletal muscle increase 1.53%. BMI decrease 2.58 and visceral fat area reduce 17 cm^2^. The symptoms of urine leakage were completely relieved. After 1 h pad test: 0.1 g. Kupperman score: 28 points.

### Case 3

A 55-year-old postmenopausal woman found urinary leakage for more than 10 years. She had two vaginal deliveries, and both of two delivery processes were smooth. The weight of the fetus was unknown, and she was not assisted by forceps. After giving birth to the second child, slight cough and trotting can lead to leakage of urine, without symptoms of frequent urination, urgency and pain. The urine leakage was 9.6 g after 1 h urine pad test. Height: 162 cm, Weight: 73.7 kg, BMI: 28.08. Pelvic examination: no obvious prolapses were observed in uterine and vaginal walls. Kupperman score: 24 points. Treatment: 4 weeks of ketogenic diet treatment. After 4 weeks of ketogenic diet treatment, the results are shown in the Figs. 1 and 2 and Table 1 below. The weight loss is 6.2 kg, the percentage of body fat decrease by 4.7%, and proportion of body skeletal muscle increase 2.89%. BMI decrease 2.32 and visceral fat area reduce 30.5cm^2^. Self-conscious leakage of urine is obviously better than before. One hour urine pad test: 4.5 g. Menopausal symptoms improved significantly and Kupperman score was 13 points.

### Case 4

A 55-year-old postmenopausal woman was diagnosed with SUI for 20 years. In 1990, she underwent cesarean delivery because of the failure of induced labor, and gave birth to a baby with a fetal weight of 3650 g and no postpartum hemorrhage. It has been 20 years since she developed symptoms of cough and trotting to leak urine. She has no symptoms of frequent, urgent and painful urination.The urine leakage was 7.3 g after 1 h urine pad test. Height: 153 cm, Weight: 66.8 kg, BMI: 28.53. Pelvic examination: no obvious prolapses were observed in uterine and vaginal walls. Kupperman score: 10 points. After 4 weeks of ketogenic diet treatment, the results are shown in the Figs. 1 and 2 and Table 1. She lost 5.2 kg of weight and 0.7% of body fat. Her BMI decrease 2.22 and visceral fat area reduce 6 cm^2^, and proportion of body skeletal muscle increase 2.54%. Self-conscious leakage of urine is obviously better than before. After one-hour urine pad test: 2.2 g. Menopausal symptoms are obviously improved, and Kupperman score is 5 points.

### Case 5

A 56-year-old postmenopausal woman found stress urinary incontinence for 30 years.

In 1989, a cesarean section was performed for cervical dystocia. The weight of the newborn was 3600 g, and there was no postpartum hemorrhage. Stress urinary incontinence occurred 20 years ago, which affected her quality of life. The results of her one-hour leak test is 3.3 g. Height: 152 cm, weight: 65 kg BMI: 28. There was no prolapse of the anterior and posterior walls of uterus and vagina. Kupperman score: 17 points. Intervention: After 4 weeks’ treatment with ketogenic diet, the results are shown in the Figs. 1 and 2 and Table 1. Results of the examination are as follows: body weight: 59.7 kg, body weight reduction: 5.3 kg, body fat percentage reduction: 2.5%, proportion of body skeletal muscle increase 1.37%. BMI reduction: 2.32 and visceral fat area reduction 10 cm^2^. Self-conscious that there is no urinary leakage. After one-hour urine pad test: 0.4 g, menopausal symptoms are obviously improved, and kupperman score is 12 points.

## Discussion and conclusion

SUI is a common disease in women. The International Consultation on Incontinence and National Institute for Health and Clinical Excellence suggest that patients with urinary incontinence should be treated first by conservertive treatment. For obese women with SUI, weight loss is the first-line treatment, but weight loss has always been a difficult problem for obese women. Although traditional diet combined with exercise can reduce weight, the effect of weight loss is slow, and the patient's compliance is poor. Ketogenic diet is an efficient method of body mass management. It can quickly loss weight and has achieved significant results in the treatment of obese women with polycystic ovary syndrome [9, 12]. Previous results have shown that ketogenic diet can improve metabolic syndrome, reduce insulin resistance and fasting blood glucose, improve ovarian function, and help women with PCOS regain their menstrual cycles [12, 13]. In these cases, the ketogenic diet is first applied to the treatment of elderly obese female patients with stress urinary incontinence, and it is found that the ketogenic diet could effectively reduce body weight, improve the symptoms of urine leakage, and improve menopausal symptoms. We believe that the mechanism of its treatment may be the result of improving multiple factors affecting stress urinary incontinence and the interaction of various factors.

Abdominal pressure is one of factors affecting urination.With the decrease of estrogen, the body fat weight increases in the postmenopausal elderly women, and the incidence of central obesity is increased. Previous epidemiological studies have confirmed the correlation between obesity and SUI. The NHS study (a prospective cohort study, n = 83,355, aged 37–54) confirmed that overweight women has a significantly increased risk of urinary incontinence, with an increase in body fat mass of 1 kg and a 3% risk increase. For every increase in BMI of 1 kg/m^2^, the risk increased by 7% [6]. Therefore, weight loss can effectively improve stress urinary incontinence, and only a 5%-10% reduction in body mass can significantly improve the symptoms of SUI [14]. In 2020, the American College of Obstetrics and Gynecology reviewed 39 studies of 43 populations and found that diet and exercise intervention could reduce the incidence of stress urinary incontinence by 15–18%, and pointed out that the long-term management of obesity could further reduce the incidence of urinary incontinence [15]. Therefore, weight loss is the first-line treatment for stress urinary incontinence in obese women. In these cases, four weeks of ketogenic diet treatment can significantly reduce body weight, reduce the visceral fat area, and improve the symptoms of urine leakage and the amount of urine leakage. Therefore, the results of these cases suggest that ketogenic diet can quickly reduce body weight and intra-abdominal obesity, thereby reducing intra-abdominal pressure and the motivation for the occurrence of SUI.

Urethral closure pressure and the location of the proximal urethra are also one of the factors that affect SUI. Under normal circumstances, there is a rich blood supply between the smooth muscle of the urethra and the inner epithelial layer, which can thicken the mucosa and cause the natural closure of the urethral wall, thus improving the urethral static pressure. The urethral epithelial mucosal vascular plexus is sensitive to estrogen, which can make the mucosal blood flow rich, soft and thick. In the absence of estrogen, poor blood supply at the submucosa can affect the tight closure of the urethra. In postmenopausal women, urethral closure pressure decreases with decreasing estrogen. A slight increase in abdominal pressure can exceed the urethral closure pressure, and urinary incontinence occurs. The patient in these cases are early menopausal women. During the ketogenic diet treatment, menopausal symptoms are significantly improved. Our previous research on women with polycystic ovary syndrome found that ketogenic diet can improve ovarian function, improve AMH level and restore menstrual cycle [9]. These results suggest that ketogenic diet can improve women’s ovarian function. For perimenopausal women, the ovarian function declines, a series of perimenopausal symptoms appear, and Kupperman score increases, which means the severity of climacteric symptoms. This study found that ketogenic diet can significantly reduce Kupperman score. Therefore, we speculate that ketogenic diet can improve ovarian function and increase urethral closure pressure and improving the symptoms of urinary incontinence. In addition, with the decline in pelvic floor function of aging women, the weak pelvic floor tissues, including the weakening of pelvic floor muscle strength, cause the proximal urethra to drop to a lower position. When the abdominal pressure increases, the pressure cannot be uniformly transmitted to the bladder and the proximal end of the urethra, but is more transmitted to the bladder, which causes the internal pressure of the bladder to exceed the closure pressure of the urethra, and urinary incontinence occurs. These cases found that during the ketogenic diet treatment, the patient was given sufficient high-quality protein, protein rich in branched-chain amino acids, etc. through nutrition management. And we found that the proportion of body skeletal muscle was significantly increased, the body fat rate was significantly decreased. Based on the previous research results, we believe that the ketogenic diet can effectively reduce the body fat rate and improve the proportion of body skeletal muscle. Therefore, we speculate that the ketogenic diet may improve the pelvic floor muscle strength and restore the urethral position, thereby reducing the occurrence of stress urinary incontinence. However, this speculation still needs to be confirmed by subsequent studies.

In summary, this is the first report on the therapeutic effect of ketogenic diet on SUI in obese women. It is found that ketogenic diet can significantly improve urine leakage, reduce body weight, decrease visceral fat area, improve proportion of body skeletal muscle, and reduce BMI. Its mechanism may be related to the reduction of abdominal pressure and the restoration of the location of the urethra. However, its mechanism still needs further researches to confirm. This study is an observational study of 5 cases reports, with a small number of cases, so there are some limitations. In the future, we hope to verify the effectiveness of ketogenic diet in the treatment of stress urinary incontinence through prospective randomized controlled studies. This report reveals that ketogenic diet may become one of the effective methods for the treatment of stress urinary incontinence in obese women in the future, providing a minimally invasive, highly profitable and highly compliant treatment for stress urinary incontinence in obese women.

## Data Availability

The data that support the findings of the study are available from the corresponding author upon reasonable request.
